# Evaluation of a liquid-phase colorimetric method for rapid antibacterial susceptibility testing

**Published:** 2013-09

**Authors:** Siavosh Salmanzadeh-Ahrabi, Tanıl Kocagöz

**Affiliations:** 1 Department of Biological Sciences, Alzahra University, Tehran, Iran; 2 Department of Medical Microbiology, Acibadem University, School of Medicine, Istanbul, Turkey

**Keywords:** Disk diffusion, susceptibility, rapid test

## Abstract

**Background and Objective:**

Early determination of antibacterial susceptibility increases the success of therapy, decreases unnecessary use of antibacterial agents and side-effects, and lowers the overall healthcare cost.

**Materials and Methods:**

A rapid colorimetric method for antibacterial susceptibility testing named Qui-Sensitest (Inovative Biotechnology Organization, Istanbul, Turkey) was evaluated in this study.

**Results:**

Qui-Sensitest proved to be a reliable rapid test for determining antibacterial susceptibility having an overall agreement of 97.6% with Kirby Bauer disk diffusion test results for enteric bacteria with 0.4% of major discrepancies and 2.0% of minor discrepancy.

**Conclusion:**

Since the test makes the results available in 3.5-6 hours, it can provide the means for choosing the right treatment regimen the same day the infectious agent is isolated.

## INTRODUCTION

**Fig. 1:**
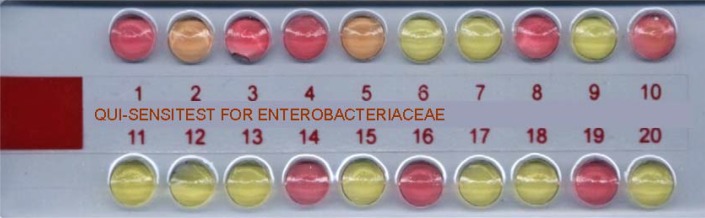
Qui-Sensitest strip, inoculated by a clinical isolate, as seen after 4 hours of incubation. Wells from 1 through 18 contain various antibacterials as listed in Table 1. Red: Resistant; Orange:Intermediate; Yellow: Susceptible. Well no 19 is growth control and no 20, medium control.

Early determination of antibacterial susceptibility of bacteria, isolated from cases like meningitis, bacteremia and sepsis is crucial for the selection of convenient therapy as soon as possible. Even when there is no such urgent need, determination of antibacterial susceptibility on the same day the culture results are obtained, is necessary for the selection of the right treatment regimen, for increasing the success of therapy, for lowering the rate of side-effects and mortality, and for cutting down the healthcare costs (1–4). We have evaluated a test called Qui-Sensitest (Trends in Inovative Biotechnology Organization –TIBO-, Istanbul, Turkey) for its efficiency in early determination of antibacterial susceptibility results. Qui-Sensitest is a strip test containing a special medium which changes its color due to metabolic activity of growing bacteria (Fig. 1). In this test, antibacterials and the concentrations to be tested for each group of organisms are selected according to the CLSI guidelines (5). In this study Qui-Sensitest strips for enterobacteriaceae (Fig. 1) were used to determine susceptibility of enteric bacteria, isolated from blood of bacteremic patients, to 18 different antibacterials and the results were compared to those obtained by Kirby Bauer disk diffusion method.

## MATERIALS AND METHODS

### Clinical isolates

To evaluate Qui-Sensitest we studied frozen stocks of blood isolates of bacteremic patients, since these patients represented one of the groups that should urgently be administered the appropriate antibiotics. One hundred isolates belonging to enterobacteriacea (48 *Escherichia coli*, 23 *Klebsiella pneumoniae*, 9 *Enterobacter aerogenes*, 6 *Serratia marcescencs*, 5 *Klebsiella oxytoca*, 3 *Enterobacter agglomerans*, 2 *Proteus mirabilis*, 1 *Enterobacter cloaca*, 1 *Citrobacter freundii*, 1 *Salmonella typhi*, 1 *Salmoella paratyphi A*) grown from blood samples of bacteremic patients were included in the study. When there were more than one isolate from different samples of the same patient, only one was included in the study.

### Susceptibility determination by Qui-Sensitest

Qui-Sensitest was performed according to manufacturer's protocol. Firstly, 50µl of the liquid special medium was put in the last well of the test strip as the media control. Then a few colonies from freshly overnight grown culture plates which had been prepared from the frozen stocks of blood isolates were suspended in Qui-Diluent to produce approximately a turbidity of 0.5Mc Farland. From this suspension, 100µl was transferred into the special medium. After mixing, 50µl was dispensed into the wells from number 1 to 19. The 19th well which did contain any antibacterial was growth control well. The plates were incubated at 35°C until the color of the media in the growth control well turned from yellow to red. It was assured that there was no color change in the media control well at the end of incubation. The tested organism was evaluated as resistant to a certain antibacterial if the color of the medium turned from yellow to red in the well that contained this antibacterial or susceptible if it stayed yellow. Orange color indicated intermediate susceptibility (Fig. 1).

### Susceptibility determination by Kirby Bauer disk diffusion method

From the bacterial suspensions prepared for Qui-Sensitest, bacteria were spread on the surface of Mueller Hinton agar and paper disks containing the same antibacterials used in Qui-Sensitest. The plates were incubated for 18-24 hours at 35°C. The inhibition zone diameteres were measured and interpreted according to the CLSI standards (5).

### Evaluation of the results

Qui-Sensitest was evaluated by comparing the susceptibility results with those of Kirby Bauer disk diffusion test. If the test results were the same, either susceptible or resistant by both tests, it was defined as “agreement”. If the result was susceptible or resistant by one test and intermediate with the other, this was called “minor discrepancy”. If the result was susceptible by one test and resistant by the other it was called “major discrepancy”.

## RESULTS

The susceptibility results of 100 blood isolates belonging to enterobacteriaceae, with Qui-Sensitest were obtained in 4 hours for the majority of the strains, the range being from 3.5 to 5 hours. The agreement between the susceptibility results obtained by Qui-Sensitest and Kirby Bauer disk diffusion test, for 18 different antibacterials in enteric bacteria that caused bacteremia is shown in Table 1. The total agreement, between two methods, (obtained by comparing 1800 individual antibacterial tests), was 97.6%. The agreement ranged between 94% and 100% when antibacterials were evaluated individually. The overall major discrepancy was 0.4% and minor discrepancy was 2.0%.

**Table 1:** Antibacterial content of Qui-Sensitest strips and the percentage of agreement between Qui-sentitest and Kirby Bauer disk diffusion susceptibility results for 100 enteric strains that caused bacteremia.

Antibacterial	Final Concentration(µg/ml)	Minor Discrepancy(%)	Major Discrepancy(%)	Total Discrepancy(%)	Total Agreement(%)
Amikacin	24	2	0	2	98
Ampicillin	16	0	2	2	98
Aztreonam	16	2	0	2	98
Cefalothin	16	3	0	3	97
Cefazolin	16	3	0	3	97
Cefotaxime	24	5	1	6	94
Ceftazidime	16	0	0	0	100
Ceftriaxone	24	4	0	4	96
Cefuroxime	16	1	1	2	98
Ciprofloxacin	2	1	0	1	99
Gentamicin	6	3	1	4	96
Meropenem	8	0	0	0	100
Ofloxacin	4	0	0	0	100
Piperacillin	48	4	0	4	96
Ampicillin/Sulbactam	16/8	3	1	4	96
Cefoperazone/Sulbactam	32/8	4	0	4	96
Trimethoprim/Sulfamethoxazole	4/76	0	1	1	99
Tobramycin	6	1	0	1	99
Total		2.0	0.4	2.4	97.6

## DISCUSSION

Clinical and financial benefits of early reporting of antibacterial susceptibility results have been shown in many studies (1–4). Barenfanger *et al*. reported that early reporting of antibacterial susceptibility test results decreased the length of stay in the hospital by 2.0 days and the average total cost for patient by $2395 (1). In another group of patients Doern *et al*. reported a cost saving of $4194 per patient and additionally a statistically significant lower mortality rate in rapid antibiotic susceptibility test group (2). In recent years, major technological advances have been made in clinical microbiology that have resulted in rapid reporting of antimicrobial susceptibility results that many regard as the most important information generated by the microbiology laboratory (3). Although several automated systems aiming to provide early antibacterial susceptibility results became available, only limited information about the accuracy and especially the speed of these systems can be found in literature. In comparative evaluations of susceptibility testing procedures, very major errors should occur in < 1.5% of all tests, and the overall agreement between tests and the reference method should be 95% (6).

Vitek (bioMerieux, NC, USA) and MicroScan Walkaway (Diamond Diagnostics, MA, USA) are two of the most commonly used automated antimicrobial susceptibility test systems. A study evaluating susceptibility of Gram-negative bacilli to eleven antibacterials using MicroScan Rapid Neg MIC/Combo panels (Diamond Diagnostics, MA, USA) and auto SCAN-W/A (Baxter MicroScan, West Sacramento, CA) showed that the results were available between 3.5 and 7.0 hours in 92.7% of the isolates and overall agreement with the standard test was 94% with a 3.4% major error rate (7). McGregor *et al*. evaluated Microscan and found out very major or major discrepancies in 2% and minor discrepancies in 8% of Gram-negative susceptibility tests, the results being available in 7 hours for 93% of the isolates (8). Comparison of Vitek and Cobas Micro Systems, (Roche Diagnostics, Basel, Switzerland) with a semi-automated conventional microsystem MIC2000 (Dynatech, McLean, Va., USA), for susceptibility testing of Gram-negative bacilli revealed 86% overall agreement with 3% major discrepancies for Vitek and 90% overall agreement with 2% major discrepancies for Cobas Micro systems (9). Evaluation of 500 Gram negative isolates to determine the number of major susceptibility interpretation discrepancies between the Vitek and MicroScan Walkaway for 9 antimicrobial agents revealed only 1.06% discrepancies between these tests (10).

Ling *et al*. compared susceptibility testing results of 228 various members of the enterobacteriaceae, *P. aeruginosa* and other Gram-negative bacteria, obtained with the Vitek 2 AST-No; 12 cards with those obtained by the broth microdilution method. They have reported 0.5% major errors (resistant with the Vitek 2 system but sensitive by the broth microdilution method) and 0.4% very major errors (sensitive with the Vitek 2 system but resistant by the broth microdilution method) (11).

In this study Qui-Sensitest proved to be a reliable rapid test for determining antibacterial susceptibility of enterobacteriaceae having an overall agreement of 97.6% with Kirby Bauer test results for enteric bacteria with only 0.4% major discrepancies. Since the test make the results available between 3.5 and 6 hours, it may have a significant impact on lowering length of stay in the hospital, total cost for patient care and even mortality by providing the means for choosing the right treatment regimen the same day the infectious agent is grown. In serious cases the susceptibility results may be confirmed by conventional standard tests.

This is the first study evaluating Qui-Sensitest for its speed and efficiency in correctly identifying antibacterial susceptibility. Further studies are needed in different settings to reveal if this novel rapid system can be used as a reliable method.
